# Composition, Anti-Diabetic, and Antioxidant Potential of *Raphanus sativus* Leaves

**DOI:** 10.3390/molecules29235689

**Published:** 2024-12-01

**Authors:** Dominika Kajszczak, Dorota Sosnowska, Barbara Frąszczak, Anna Podsędek

**Affiliations:** 1 Institute of Molecular and Industrial Biotechnology, Faculty of Biotechnology and Food Sciences, Lodz University of Technology, Stefanowskiego 2/22, 90-537 Łódź, Poland; dominika.kajszczak@p.lodz.pl (D.K.); dorota.sosnowska@p.lodz.pl (D.S.); 2 Department of Vegetable Crops, Poznań University of Life Sciences, Dąbrowskiego 159, 60-594 Poznań, Poland; barbara.fraszczak@up.poznan.pl

**Keywords:** leaves, red radish, starch digestion, α-amylase, α-glucosidase, AGEs

## Abstract

Limiting and/or slowing down the starch digestion process and consequently the release of glucose can be an important strategy for the prevention of type 2 diabetes (T2D). The aim of the current in vitro study was to assess the anti-diabetic and antioxidant potential of red radish leaves of the Carmen, Jutrzenka, Saxa, and Warta cultivars. In the context of anti-diabetic activity, the effect of leaves on potato starch digestion and free glucose binding, as well as inhibitory effects of leaf extracts against α-amylase and α-glucosidase and non-enzymatic glycation (AGEs) were determined. The basic chemical composition, quantitative composition of phenolic compounds, and antioxidant activity of leaves were also estimated. This study showed that all radish leaves inhibited the breakdown of potato starch and showed their ability to bind glucose. This activity was correlated with the content of hydroxycinnamic acids, protein and dietary fiber while flavones was probably responsible for glucose binding. Leaf extracts inhibited α-glucosidase activity and formation of AGEs but were practically inactive towards α-amylase. Inhibition of α-glucosidase activity was related to the content of proanthocyanidins and inhibition of AGEs formation to flavonols. These results point to radish leaves, especially the Warta and Jutrzenka cultivars, as a potential natural remedy for treating T2D.

## 1. Introduction

Diabetes is the most prevalent chronic metabolic disease worldwide, and its incidence is increasing at an alarming rate. Currently, according to the International Diabetes Federation, 540 million of the adult population (20–79 years old) suffer from diabetes, and the number of patients is estimated to increase by 46% by 2045 [1]. The major form of diabetes, formerly known as non-insulin dependent diabetes, i.e., type 2 diabetes (T2D), accounts for 90–95% of reported diabetic cases. T2D is characterized by chronic hyperglycemia since the body becomes resistant to the normal action of insulin, produces excess insulin, and ultimately causes β-cells in the pancreas to wear out. Currently, several anti-diabetic drugs are used, but they have several limitations on efficacy and safety to combat this disease [2,3,4]. They exhibit side effects such as gastrointestinal symptoms, impaired function pancreatitis, heart failure, edema, and weight gain, etc. Therefore, the search of potent and safe drugs from nature is a great challenge of clinical medicine. More than 200 plant species have been found to exhibit potential benefits in the prevention of diabetes, and some of them could be utilized in the treatment of diabetes as safe and low-cost drugs [3,4,5,6,7]. Apart from medicines, diet rich in plant-derived food also plays a very important role in management of diabetes [8,9,10].

In vivo studies have revealed that different morphological parts of *Raphanus sativus* L. (family Brassicaceae), known as radish, has been employed as an effective remedy for diabetes. Radish microgreens significantly reduced blood glucose level and improved the liver and kidney parameters in streptozotocin (STZ)-induced-diabetic rats [11]. Other studies have shown that the administration of radish microgreens to diabetic and/or aflatoxicated rats improved insulin sensitivity and function parameters of liver and kidney and decreased insulin resistance [12]. Radish seeds decreased hyperglycemia via reducing insulin resistance, limiting intestinal glucose absorption, and increasing glucose uptake in skeletal muscles [13]. In addition, an extract of radish roots reduced blood glucose in glucose-loaded rats, but no significant differences were seen in cholesterol and triglycerides levels, and it did not affect kidney and liver function [14]. Moreover, radish leaf extract reduced nuclear factor kappa B (NFkB) expression in the heart muscle in diabetic Wistar rats [15]. In vitro studies have shown an inhibitory effect of radish sprout extract on α-glucosidase activity [16] and that radish microgreen as well as mature leaf extract had a substantial impact on α-amylase [17,18]. Furthermore, red radish roots fried in sunflower oil slowed down starch hydrolysis from pasta during in vitro digestion process [19].

Radish (*Raphanus sativus* L.) is a popular root vegetable consumed worldwide [20]. The radish roots are characterized by different colors, sizes, and shapes and can be consumed raw, cooked, dried, or preserved. Radish leaves are not typically eaten, and therefore they constitute agro-industrial as well as household by-products. Some studies revealed that the nutritional value of radish leaves far exceeded the corresponding value for roots due to the higher content of protein, ash, dietary fiber, and ascorbic acid [21,22]. Additionally, the leaves were richer in phenolic compounds and showed higher antioxidant activity than roots. Few studies indicate the antioxidant, anti-inflammatory, and antimicrobial of radish leaf extract [15,17,23]. Thus, radish leaves have been considered as interesting research material in the context of human health benefits, including people with type 2 diabetes. Moreover, use of by-products from plant-derived food is currently of much interest, both from waste reduction and product value addition points of view.

Despite these considerations, to the best of our knowledge, the inhibitory potential of radish leaves with respect to starch hydrolysis, α-glucosidase activity, as well as the formation of glycation end products (AGEs) were not determined. Thus, the aim of this study was to investigate the effects of red radish leaves from four plant cultivars (Carmen, Jutrzenka, Saxa, and Warta) on the course of starch decomposition under in vitro simulated digestion. Moreover, it was checked whether the radish leaf extract has an influence on the activity of starch-hydrolyzing digestive enzymes and the formation of AGEs. In addition, radish leaves were characterized in terms of their basic chemical composition, composition of phenolic compounds, and antioxidant activity.

## 2. Results

### 2.1. Proximate Composition of Radish Leaves

The proximal composition of four cultivars of radish leaves is presented in Table 1. Significant differences (*p* < 0.05) between the four analyzed cultivars were demonstrated only in relation to fat content. Radish leaves were dominated by carbohydrates (48.18–49.98 g/100 g dw) and fiber (41.07–44.39 g/100 g dw), while organic acids were present in the lowest concentration (0.71–0.86 g/100 g dw). The composition of organic acids is presented in Table S1, and the results demonstrated succinic, citric, and oxalic acids as the main radish leaf components. The radish leaves of the Warta cultivar were characterized by the highest content of protein, carbohydrates, organic acids, and dietary fiber with insoluble fractions. The Carmen cultivar exhibited the highest ash and soluble dietary fiber content, while the Jutrzenka cultivar had the highest fat content. Moreover, the dominant component of fiber was the insoluble fraction, which accounted for over 95% of its content.

### 2.2. Bioactive Compound Content and Antioxidant Activity of Radish Leaves

The contents of radish leaf secondary metabolites with antioxidant activity are summarized in Table 2. L-ascorbic acid content was determined by chromatographic method, while the remaining compounds were analyzed by spectrophotometric methods.

Statistically significant differences were found for the cv. Warta leaves in terms of the content of total proanthocyanidins and for cv. Saxa and Carmen in relation to the content of total phenolics. Quantitatively, phenolic compounds dominated in all radish leaves tested, followed by L-ascorbic acid and proanthocyanidins. In the group of photosynthetic pigments, the content of total chlorophyll was on average ten times higher than the content of total carotenoids. In addition, chlorophyll *a* dominated in all samples with twice the content than chlorophyll *b*. Regarding the radish cultivar, cv. Warta and cv. Saxa exhibited the highest contents of proanthocyanidins and chlorophyll *b*, respectively. The remaining bioactive compounds were found in the highest concentrations in the leaves of the Jutrzenka cultivar.

The presence of L-ascorbic acid (vitamin C), phenolic compounds, as well as chlorophyll and carotenoid pigments determine the antioxidant properties of radish leaves. Their antioxidant activity was determined by four methods, such as scavenging activity toward ABTS^•+^ cation radical (ABTS), superoxide anion radical (SARSA), as the potential to reduce ferric ion to ferrous ion (FRAP), and ferric-ion-chelating activity (FCA). The results are presented in Table 3.

All methods indicated the highest antioxidant capacity for leaves of radish cv. Jutrzenka. On the contrary, the leaves of the Carmen cultivar were characterized by the lowest antioxidant capacity in the ABTS, FRAP, and FCA methods and the leaves of the Saxa cultivar in the SARSA method. Similar antioxidant activity (no statistically significant differences) was observed for the leaves of the Jutrzenka and Warta cultivars in the ABTS and FRAP methods and for the Saxa and Warta cultivars in the FCA method. Considering the results for the SARSA method, the efficiency of scavenging the superoxide anion radical by the leaves of the Warta cultivar was like the activity of the Carmen and Saxa leaves.

### 2.3. Compsition of Phenolic Compounds of Radish Leaves

The data about phenolic profile of four cultivars of radish leaves are summarized in Table 4. The tentative identification of phenolic compounds based on fragmentation patterns and comparison with literature data are presented in Table S2, and their chromatograms are demonstrated in Figure S1 [19,24,25,26,27,28,29,30]. A total of twenty-two phenolic compounds were identified in the radish leaves, including hydroxycinnamic acids, flavonols, flavones, and anthocyanins. It is worth emphasizing that there are no anthocyanin pigments in the leaves of the Jutrzenka cultivar. The data presented in Table 4 indicate the cultivar differentiation of radish leaves in terms of phenolic profile. The largest number of compounds, as many as nineteen, were identified in the Warta cultivar, followed by seventeen in the cv. Jutrzenka, sixteen in cv. Saxa, and only fifteen compounds in the Carmen cultivar. Leaves of the radish cv. Warta were characterized by the highest content of hydroxycinnamic acids and flavones. On the other hand, the leaves of the Jutrzenka cultivar contained the largest amounts of flavonols and total phenolics. From the quantitative point of view, flavonols were the most abundant phenolic compounds class, representing from 70% in the cv. Warta to 83% in the cv. Jutrzenka of total phenolic compounds.

The identified flavonols were kaempferol derivatives in the form of glycosides and acylated glycosides with *p*-coumaric acid. The most representative flavonol in the leaves of the three radish cultivars was kaempferol 3-*O*-coumaroyl glucoside (282.62–429.84 mg/100 g dw), except for cv. Jutrzenka, where kaempferol 3-(*p*-coumaroyl) sophorotrioside was present in the highest amount (502.53 mg/100 g dw). It was found that among seven phenolic acids identified, caffeic acid glucoside occurred in the largest amounts in the leaves of the Jutrzenka and Carmen cultivars (44.46 and 6.49 mg/100 g dw, respectively). Contrarily, *p*-coumaric acid (72.84 mg/100 g dw) and feruoylmalic acid (30.95 mg/100 g) were the predominant compounds in the leaves of Warta and Saxa cultivars, respectively. Among two identified apigenin derivatives (flavones), the content of apigenin-7-*O*-rutinoside ranged from 180.16 to 296.82 mg/100 g dw and was the highest in the leaves of the Warta variety. The concentration of the second flavone (apigenin-C-hexoside-C-pentoside) was much lower (1.74–5.28 mg/100g dw). The identified anthocyanins were mono- or diacylated derivatives of pelargonidin 3-diglucoside-5-glucoside with pelargonidin-3-(feruloyl)diglucoside-5-(malonyl) glucoside as the most abundant (4.10–6.61 mg/100 g dw). Significant differences (*p* < 0.05) between the four analyzed cultivars were demonstrated in relation to the sum of flavonols and total phenolics, caffeic acid glucoside, ferulic acid glucoside, 1,2-disinapoylglucoside, feruoylmalic acid, and kaempferol 3-(*p*-coumaroyl)sophorotrioside.

### 2.4. Effect of Radish Leaves on Starch Hydrolysis

**Figure 1:**
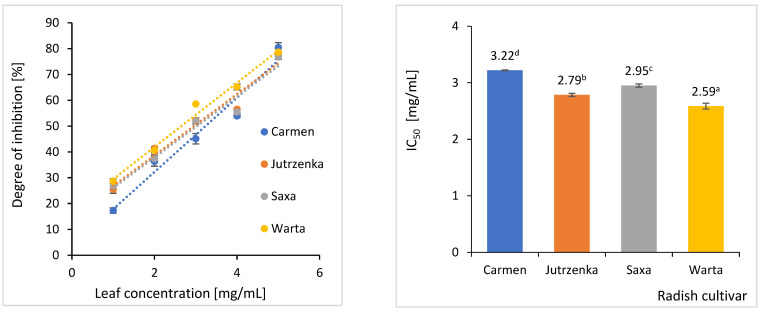
Inhibition of glucose released after 120 min simulated potato starch digestion in the presence of freeze-dried leaves from *Raphanus sativus*. This Figure shows mean value ± standard deviation (*n* = 3). The means with different letters superscripts differ statistically at *p* < 0.05.

Simultaneous digestion of potato starch and freeze-dried radish leaves for 2 h resulted in a reduction in the amount of glucose released (Figure 1). The degree of starch hydrolysis inhibition increased with the dose of leaves in the digested sample. The highest inhibitory activity (IC_50_ = 2.59 mg/mL) was observed for leaves of the Warta cultivar. For comparison, the inhibitory activity of the least effective cv. Carmen leaves was 24% lower.

It was also checked whether the components of radish leaves could bind glucose, which is released during the digestion of carbohydrates. The glucose absorption capacity of the radish leaves is presented in Table 5.

The glucose-binding capacity of the freeze-dried radish leaves was dependent on the radish cultivar and the glucose concentration. Radish leaves cv. Warta exhibited the highest increase in glucose binding with increasing glucose concentration in the solution from 10 mM to 50 mM (2.1–fold), while changing the glucose concentration from 10 mM to 100 mM resulted in the highest increase in binding activity (3.5–fold) for cv. Saxa leaves. Among the four radish cultivars tested, the highest glucose-binding capacity was found for the leaves of the Warta and Carmen cultivars.

To understand which radish leaf components were responsible for the demonstrated activity, Pearson correlation analyses were performed between inhibitory effect of starch digestion or glucose-binding capacity, and the composition of macronutrients and phenolic compounds (Table S3). High correlation coefficients (*r* > −0.93) indicated a significant effect of total phenolics determined using a Folin–Ciocalteu method and hydroxycinnamic acids on inhibition of potato starch digestion. Moreover, correlation coefficients in the range of −0.78 to −0.86 suggest dependence of the degree of starch hydrolysis on the content of both total fiber and its insoluble fractions, protein, and total phenolics determined using a UPLC method. Contrarily, the above radish leaf components were only slightly responsible for glucose binding, whereas the glucose-binding capacity was correlated with the content of flavones (*r* = 0.932) and the level of proanthocyanidins (r = 0.762). In the case of the soluble dietary fraction, a negative correlation was observed with both starch digestion and glucose-binding capacity.

### 2.5. Effect of Radish Leaf Extracts on α-Amylase and α-Glucosidase Activity

Phenolic compounds are postulated to be active inhibitors of various enzymes; therefore, the effectiveness of glycoside hydrolase inhibition by radish leaf extracts isolated from four radish cultivars using 80% ethanol, and, in the final stage, acetone was evaluated. The extracts were characterized in terms of the content of various groups of phenolic compounds using the UPLC-MS method (Table 6), the exact composition of which is presented in Table S4. Flavonols were the most abundant class of phenolic compounds (74.20–104.96 mg/g) of the leaf extracts and represented from 70% in the cv. Warta to 83% in the cv. Jutrzenka of total phenolic compounds, followed by flavones with a 10–19% contribution in the sum of phenolics. For comparison, the content of proanthocyanidins did not exceed 0.22 mg/g, and the content of anthocyanins was below 0.90 mg/g. Significant differences (*p* < 0.05) between the four analyzed cultivars were demonstrated only in relation to total hydroxycinnamic acids.

**Figure 2:**
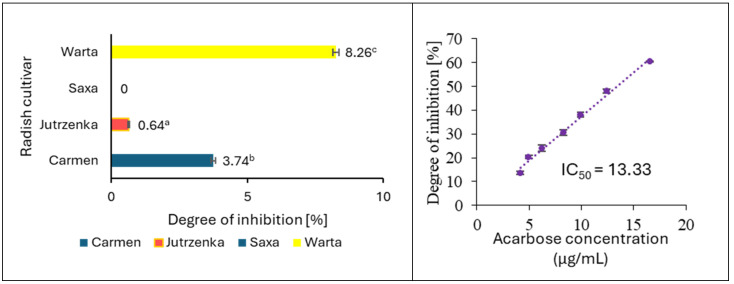
Percentage inhibition (%) of α-amylase activity in the presence of leaf extracts (7.5 mg/mL of reaction mixture) and acarbose. This Figure shows mean value ± standard deviation (*n* = 3). The means with different letters superscripts differ statistically at *p* < 0.05.

**Figure 3:**
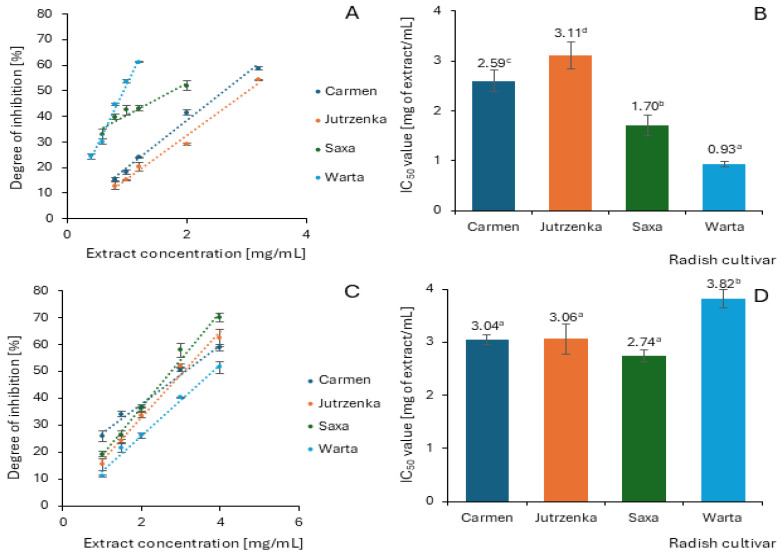
Inhibitory effects of radish leaf extracts on α-glucosidase activity in the presence of maltose (**A**,**B**) and sucrose (**C**,**D**). This Figure shows mean value ± standard deviation (*n* = 3). The means with different letters superscripts differ statistically at *p* < 0.05.

The α-amylase assay was performed using potato starch as substrate, and the results are demonstrated in Figure 2. Among the four extracts tested, only those obtained from the Warta and Saxa cultivars showed slight inhibitory activity with the degree of inhibition equal to 8.26 and 3.74%, respectively. The remaining two extracts did not present an inhibition against pancreatic α-amylase at a concentration of 7.5 mg/mL. The radish leaf extracts activities were negligible compared to acarbose (well-known antidiabetic drug), which inhibited α-amylase activity by 50% at a concentration of 13.33 µg/mL. Contrarily, radish leaf extracts proved to be effective α-glucosidase inhibitors and inhibited this enzyme in a dose-dependent manner (Figure 3A,C). Measurement of α-glucosidase activity was carried out using maltose and sucrose as substrates. The analysis of the determined IC_50_ values indicates significant differences between the cultivars only when maltose was used as a substrate (Figure 3B,D). In relation to sucrose, only the leaf extract of the Warta cultivar differed significantly from the others. It is worth emphasizing that the extract obtained from the leaves of the Warta cultivar showed the highest efficiency of α-glucosidase inhibition in the system with maltose and the lowest efficiency in the case of using sucrose as a substrate. The extreme values of IC_50_ among cultivars, against maltose differed more than three-fold, while in comparison against sucrose they differed only by 39%. However, all leaf extracts showed weaker inhibitory activity against α-glucosidase compared to acarbose. The IC_50_ values for this compound were 0.051 µg/mL and 1.37 µg/mL in the maltose and sucrose models, respectively.

The inhibitory activity of radish leaf extracts against α-glucosidase expressed as IC_50_ was highly correlated with total proanthocyanidins (*r* = −0.921) and content of flavones (*r* = −0.854) but only in assay system with maltose as substrate (Table S5). In the model using sucrose as a substrate, positive values of the correlation coefficient indicate a decrease in the inhibitory effect of leaf extracts with increasing concentration of phenolic compounds, especially flavones and hydroxycinnamic acids.

### 2.6. Effect of Radish Leaf Extracts on AGEs Formation

**Figure 4:**
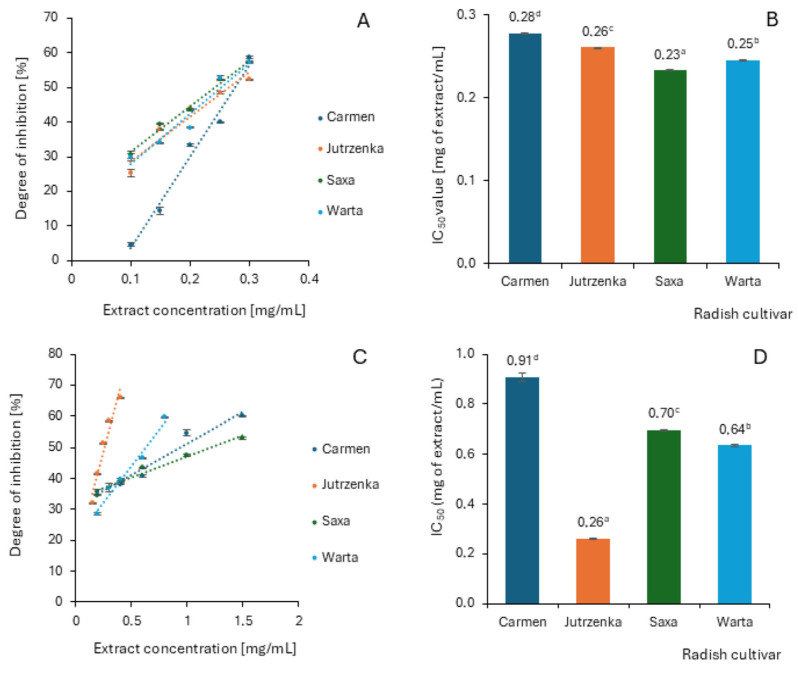
Inhibitory effect of radish leaf extracts on AGEs formation in the presence of fructose (**A**,**B**) and glucose (**C**,**D**). This Figure shows mean values ± standard deviations (*n* = 3). The values with different letters superscripts are significantly different (*p* < 0.05).

Anti-glycation activity of radish leaf extracts was evaluated through detection of fluorescent AGEs formed in the bovine serum albumin (BSA) and glucose or fructose models. The inhibition results were expressed as absolute percentages and IC_50_ value (Figure 4). The anti-glycation activity of radish leaf extracts increased with increasing concentration, indicating a dose-dependent effect. IC_50_ values within the analyzed radish cultivars differed significantly in both measurement systems. The extract obtained from the leaves of the Carmen cultivar showed the lowest efficiency of AGEs inhibition in both assays. For comparison, AGEs were inhibited to the highest extent by leaf extract from the Saxa and Jutrzenka cultivars in the BSA–fructose and BSA–glucose models, respectively. The extreme values of IC_50_ differed only by 22% and by as much as 3.5 times in assays with fructose and glucose, respectively. The most effective leaf extracts showed approximately twice as much activity as aminoguanidine. This anti-glycation therapeutic agent showed IC_50_ values equal to 0.11 ± 0.003 mg/mL and 0.14 ± 003 mg/mL in the BSA–fructose and BSA–glucose models, respectively.

The inhibitory activity of radish leaf extracts against AGEs formation expressed as IC_50_ value was highly correlated with total flavonols (*r* = −1) and content of total phenolics (*r* = −0.840) in the BSA–glucose assay system and with total proanthocyanidins (*r* = −0.720) in relation to the BSA–fructose model (Table S5).

## 3. Discussion

Plant components can act in various ways as anti-diabetic agents [3,4,5,7]. They affect the process of glucose release in the gastrointestinal tract, control of glucose absorption by up-regulation of glucose transporter, inhibit glucose production in hepatocytes, increase glucose uptake by adipose and muscle tissues, stimulate the production of insulin by the pancreas, and improve antioxidant defense. Starch is one of the main sources of energy in the human diet. Widespread preference of people to rapidly digestible starchy food in industrialized countries has been reported to be one of the reasons for hyperglycemia. Therefore, limiting and/or slowing down the starch digestion process and consequently the release of glucose can be dietary strategy for prevention of T2D [31]. This study showed that all four cultivars of radish leaves inhibited the breakdown of potato starch during in vitro processes simulating oral, gastric, and intestine digestion. In addition, significant cultivar differences were found, with the leaves of the Warta cultivar being the most effective (IC_50_ = 2.59 mg/mL), although the least effective leaves, cv. Carmen, showed only a 24% higher IC_50_ value. A previous study has shown that fried red radish roots inhibit pasta starch digestibility in vitro [19]. Co-digestion of 3.2 g of pasta with 6.8 g of radish roots resulted in a 25% reduction in the amount of released glucose. In the present study, similar inhibition values (17.31–28.72%) were obtained after adding 1.0 mg of radish leaf to 12.5 mg of potato starch. The analysis of correlation coefficients (Table S3) indicates the dependence of the starch digestion process on the presence of phenolic compounds in the leaves, especially hydroxycinnamic acids, as well as dietary fiber. Catitivelli et al. [19] found no clear relationship between total phenolic content determined by high-resolution mass spectrometry and starch hydrolysis inhibitory activity during in vitro co-digestion of pasta with red radish, cherry tomatoes, capers, dark purple eggplant, and red skin onions. This suggests that the inhibitory potential of these vegetables was related to the phenolic composition rather than to their total content. However, according to the authors, any contribution to the inhibition of starch hydrolysis of other unidentified phenolic compounds, particularly the high molecular weight ones, cannot be excluded. It should be emphasized that in our studies, the profile of phenolic compounds was determined in the undigested radish leaves, while the cited authors determined it in samples after 272 min of digestion. Phenolic compounds undergo various transformations during digestion because of pH changes and the action of digestive enzymes. Li et al. [32] found a reduction in the total anthocyanin content during the digestion of red radish roots, including the complete degradation of pelargonidin-3-diglucosie-5-glucoside and some of its mono-, di-, and tri-acylated derivatives with caffeic, *p*-coumaric, ferulic, and malonic acids. Decrease in total phenolic content and conversion of ferulic acid and chlorogenic acid to *p*-coumaric, sinapic, syringic, and/or *trans*-cinnamic acids during digestion of black radish root peel extract were also observed [33]. Similarly, in vitro gastrointestinal digestion of radish microgreens showed a decrease in the content of total phenolics and total anthocyanins [34,35]. To date, no research about the effect of in vitro digestion on radish leaf phenolic compounds has been conducted.

The digestibility of starch can be influenced by many factors, including its interactions with other components of the food matrix, including fiber, protein, lipids, and phenolic compounds [36,37,38,39,40]. For example, soluble dietary fiber components affect the digestive process by hindering the enzymatic reaction between starch and digestive enzymes by increasing the viscosity of the chyme and consequently reducing the diffusion of enzymes into the starch substrates and/or limiting the mixing efficiency. In addition, the fiber components may increase the viscosity near the brush border and thus limit the absorption of glucose released during digestion, high concentrations of which may inhibit starch hydrolysis in the digestive system [38]. In the present study, the degree of inhibition of glucose (expressed as IC_50_ value) release from potato starch during digestion was highly correlated (*r* = −0.781–−0.821) with the content of total fiber and insoluble fiber (Table S3). It should be emphasized that total fiber and their insoluble fraction constituted on average 42.52% and 41.00% of the weight of dried radish leaves (Table 1). The susceptibility of starch to hydrolysis may also be limited due to the formation of complexes with proteins and/or lipids, especially during food processing [41]. The formation of complexes between amylose and lipids reduces starch swelling capacity, its solubility in water, and susceptibility to enzymatic hydrolysis, as well as may reduce glucose absorption in the small intestine and may increase insulin response. The lipid content in the radish leaves tested ranged from 5.32 to 8.19% on a dry weight basis and was weakly correlated (*r* = −0.344) with the degree of inhibition of potato starch digestion. Starch digestibility could also be regulated by hydrogen bonds between starch and protein. Protein is found on the surface of starch granules, and it may act as a physical barrier to digestion [42,43]. Protein constituted 21.47–22.60% of the dry weight of radish leaves and their content was highly correlated (r = −0.866) with the efficiency of inhibition of enzymatic degradation of potato starch.

Many studies describe the influence of phenolic compounds on the hydrolytic decomposition of starch via the inhibition of α-amylase and α-glucosidase activity and/or the formation of inclusion and non-inclusion complexes with starch [36,37,39,44,45,46,47,48]. The presence of phenolic compounds in the diet may increase the content of resistant starch, change starch physicochemical properties, and can limit the starch accessibility to α-amylase and α-glucosidase enzymes. On the other hand, the interaction between phenolic compounds and starch may affect the stability, solubility, antioxidant activity, and bioaccessibility of these secondary metabolites. It has been shown that the phenolic hydroxyl groups in most polyphenols can form hydrogen bonds and hydrophobic and electrostatic interactions with starch molecules. The radish leaves examined in this study contained structurally diverse phenolic compounds from the group of hydroxycinnamic acids, flavonols, flavones, and anthocyanins (Table 4). The quantitatively dominant flavonols were kaempferol glycosides and their acylated derivatives with coumaric acid. Anthocyanins were represented by mono- and di-acylated pelargonidin derivatives. In addition, the leaves contained apigenin C-glycosides and hydroxycinnamic acids, mainly in combination with glucose. To the best of our knowledge, despite numerous studies on the interactions of phenolic compounds with starch and glycoside hydrolases, there are no data on acylated flavonoids. This may be partly due to the lack of commercially available standards for more complex compounds. In this context, proanthocyanidins have been better studied and were found in the radish leaves tested at a concentration of 207.87–263.94 mg/100 g dw. The results obtained by Barros et al. [49] have shown that sorghum proanthocyanidins reduce starch digestibility, increasing resistant starch content, and interact with pure amylose more strongly than with amylopectin. According to other research, procyanidins can bind to amylose independently of the starch source and can inhibit starch digestion by α-amylase in the intestine [47]. Flavanols, flavonols, flavanones, anthocyanins, isoflavones, and phenolic acids have also been reported to have such reducing effect on starch digestion [39,45,46,48]. Moreover, polyphenols can inhibit the glucose uptake from intestine lumen by direct inhibition of glucose transporter or their expression and consequently reduce postprandial glucose level [50]. The accumulation of unabsorbed glucose may also negatively affect the starch hydrolysis process [48]. The reduction in blood glucose level may also be due to the binding of glucose released in the intestine during the digestion of starch by the components of the food matrix. The radish leaves tested showed a glucose-binding capacity that depended on the glucose concentration (Table 4). This activity was highly correlated with content of flavones and proanthocyanins (*r* = 0.932 and 0.762, respectively). Other authors postulate that dietary fiber is responsible for glucose binding [51,52]. For example, the glucose absorption capacity of the extruded cocoa shell strongly correlated with soluble dietary content (r = 0.965) [51]. The above relationship was not found in the case of radish leaves because the value of correlation coefficient (r = −0.300) indicates a decrease in this activity with an increase in the content of soluble dietary fraction. On the other hand, the determined Pearson’s correlation coefficients for total dietary fiber and insoluble fraction were 0.509 and 0.486, respectively (Table S3). Similarly to our results, the glucose-binding capacity of fruit and vegetable pomace was mainly due to the presence of insoluble dietary fiber [52,53,54].

The binding interactions between phenolic compounds and α-amylase and α-glucosidase have been considered as the main reason for starch digestion inhibition. In this regard, α-glucosidase inhibitors such as acarbose and miglitol have been used to control diabetes [4]. Starch is successively digested by salivary α-amylase in the mouth, followed by pancreatic α-amylase in the small intestine to α-limit dextrins, maltotriose, and maltose. These amylolytic products are further digested by epithelial α-glucosidase with sucrase–isomatlase and maltase–glucoamylase activities to yield glucose, which is further absorbed into the hepatic portal vein via the sodium-dependent glucose transporter and glucose transport protein [38]. As displayed in Figure 2, radish leaf extracts were weak inhibitors of porcine pancreatic α-amylase with a 0.00% to 8.26% inhibition at the 7.5 mg extract/mL concentration of the reaction mixture. In regard to α-glucosidase, radish leaf extracts showed better activity with IC_50_ = 0.93–3.11 mg/mL in the assay with maltose as substrate, and with IC_50_ varied from 2.74 to 3.82 mg/mL using sucrose as substrate. Similarly to α-amylase, the inhibitory activity of the radish leaf extracts tested was negligible compared to that of acarbose (IC_50_ = 0.051 or 1.37 µg/mL depending on the type of substrate). Other studies confirmed lower inhibitory activity of radish leaves against α-amylase compared to acarbose [17,18]. The lower activity of other leaf extracts than acarbose with respect to both enzymes has also been shown by other studies [55,56]. Additionally, the results of our work suggest that the enzyme inhibitors contained in radish leaves are more active against α-glucosidase than α-amylase. According to Kwon et al. [57], weak inhibitory activity against α-amylase compared to better inhibition of α-glucosidase is very desirable because it can minimize side effects of starch digestion such as abdominal distention and flatulence.

The observed lower inhibitory activity of radish leaf extracts against both digestive enzymes tested compared to acarbose may be due to the low content of phenolic compounds (Table 6) and low or no affinity of acylated flavonols for α-amylase and α-glucosidase. The content of the above-mentioned polyphenol group in radish leaf extracts was 74.20–104.96 mg/g, which constituted 70–83% of the total content of phenolic compounds. Some studies demonstrated anti-amylase and anti-glucosidase activity of crude extract of red cabbage, which are rich in acylated cyanidin derivatives, and anthocyanin reach extract from sweet purple potato contained acylated cyanidin and peonidin derivatives [58,59]. However, the content of acylated anthocyanins of radish leaf extract was very low (0.65–0.83 mg/g), and they contributed below 0.80% in total phenolics. This may suggest their insignificant contribution to the observed inhibitory activity of the extracts, although the efficiency of α-glucosidase inhibition of leaf extracts with maltose was highly correlated (r = −0.824) with the content of anthocyanins.

Inhibitory activity of phenolic compounds against glycoside hydrolases is determined by their molecular structure [19,48,60,61]. No studies on the inhibitory activity of individual acylated flavonols are present in the literature, although many non-acylated forms have been analyzed. The results of previous studies suggest that monoglycosylation, diglycosylation, and polyglycosylation of hydroxyl groups in the flavonoid molecule may reduce their inhibitory activity against α-amylase because the hydroxyl group is essential for the inhibitory effect. In addition, the increased molecular size leads to steric hindrance for flavonoid binding to the enzyme [48]. For example, Cattivelli et al. [19] showed a weak inhibitory activity of kaempferol-3-*O*-rutinoside against α-amylase (about 20% at 500 μmol/L concentration) and a weaker inhibitory activity of quercetin diglucoside than monoglucoside. On the other hand, quercetin-4′-*O*-glucoside, quercetin-3-*O*-rutinoside, and kaempferol-3-*O*-rutinoside totally inhibited the α-glucosidase activity at this concentration. Matsui et al. [60] showed diacylated pelargonidin derivatives as stronger inhibitors of α-glucosidase activity than acylated cyanidin and peonidin derivatives. Nyambe-Silavwe and Williamson [61] demonstrated weak inhibitory activity of phenolic acids against salivary amylase and rat intestinal maltase.

In a state of hyperglycemia, glycated hemoglobin and other advanced glycation end products (AGEs) are formed, which ultimately leads to oxidative stress, altered protein activity, impaired immunogenicity, thrombosis, vasculitis, and other secondary complications of diabetes [62]. Therefore, anti-glycation has significant therapeutic importance in reducing secondary complications associated with diabetes. Crude radish leaf extracts were able to inhibit glycation process in both the BSA–glucose nad BSA–fructose models. In the BSA–glucose assay, the inhibitory activity was highly correlated with the content of flavonols and, in BSA–fructose model, with total proanthocyanidins. The multifunctional effects of different sub-groups of flavonoids, including flavonols, flavones, and anthocyanins identified in radish leaves, were summarized by Zhou et al. [63]. Generally, methylation/methoxylation and glycosilation of flavonoids might decrease the ability of aglycones. In addition, the higher IC_50_ value (42.1 µM) for apigenin-7-*O*-(acetyl)-glucopyranoside than for apigenin-7-*O*-glucopyranoside (6.8 µM) may suggest a negative effect of acylation on the inhibition of AGEs formation.

Improvement of the body’s antioxidative defense is an additional benefit of consuming radish leaves, for example, as an ingredient in salads or soups, a functional additive in other products, and in supplements form. Chronic hyperglycemia promotes the formation of free radicals, which further increases the formation of reactive oxygen species, which in turn lead to a state of oxidative stress—a key factor in the development of diabetic complications [64]. There is increasing evidence that phenolic compounds, due their antioxiadant properties, may protect cell constituents against oxidative damage [65]. Moreover, other compounds, such as vitamin C, carotenoids, and chlorophyll (Table 2), also participated in the antioxidant activity of radish leaves [66]. The antioxidant activity of the radish leaves tested was varied due to radish cultivars and the measurement system. Nevertheless, the ability of antioxidants present in radish leaves to scavenge superoxide anion radical and ABTS cation radical, as well as to reduce iron (III) ion and bind iron (II) ion, was confirmed. Other studies have also demonstrated the ability of radish leaves to scavenge peroxyl radical and synthetic DPPH radical and to reduce cupric ion [22,67].

## 4. Materials and Methods

### 4.1. Standards and Reagents

Intestinal acetone powder from rat source of α-glucosidase (EC 3.2.1.20), α-amylase from porcine pancreas type VI-B (EC 3.2.1.1), pancreatin from porcine pancreas, pepsin from the gastric mucosa of pigs, bile from bovine and ovine, D-glucose, D-fructose, sodium azide, bovine serum albumin (BSA), phosphate-buffered saline (tablet), TRIS-HCl, caffeic acid, apigenin 7-glucoside, sinapic acid, ferulic acid, ascorbic acid, citric acid, fumaric acid, malic acid, oxalic acid, succinic acid, tartaric acid, sodium chloride, maltose, sucrose, meta-phosphoric acid, methanol (LC–MS grade), and acetonitrile (LC-MS grade) were obtained from Sigma Aldrich (Steinheim, Germany). Acetone, ethanol, hydrochloric acid, sodium hydroxide, sodium bicarbonate, iodine, potassium iodide, disodium phosphate, and monosodium phosphate were purchased from Chempur (Piekary Śląskie, Poland). Potato starch, methanol, and calcium chloride were purchased from POCH (Gliwice, Poland) and glucose test from Biomaxima SA (Lublin, Poland). Formic acid (LC-MS grade) was purchased from Carl Roth (Karlsruhe, Germany). Pelargonidin 3-glucoside and *p*-coumaric acid were obtained from Extrasynthese (Lyon, France). Kaempferol 3-glucoside was purchased from PhytoLab (Vestenbergsgreuth, Germany). Ultrapure water was prepared in the laboratory using a Simplicity Water Purification System (Millipore, Marlborough, MA, USA).

### 4.2. Plant Materials

Four radish cultivars were used as experimental planting material. The seeds of these cultivars were obtained from a commercial source (“W. Legutko” Company, Jutrosin, Poland). The Carmen cultivar produces large, spherical, red roots and is a medium-early cultivar with a mild flavor. It maintains its consumption value for a long time. The Jutrzenka cultivar produces egg-shaped, pink roots. The flesh is white and has a mild taste. The Saxa 2 cultivar forms spherical roots with a red color. It is an early, high-yielding cultivar. The Warta cultivar produces cylindrical scarlet-red roots with white, mild-tasting flesh. The plants were cultivated in the open field at the ‘Marcelin’ Research Station, Poznań University of Life Sciences, Poland. The seeds were sown using the line sowing method. The seeds were sown maintaining plant spacing of 20 cm × 5 cm. The plants were collected 35 days after sowing. Leaves were dried by lyophilization (Martin Christ, Freeze Dryer, Alpha 1-2/LD, Osterode am Harz, Germany) for 48 h.

### 4.3. Preparation of the Extracts

**Figure 5:**
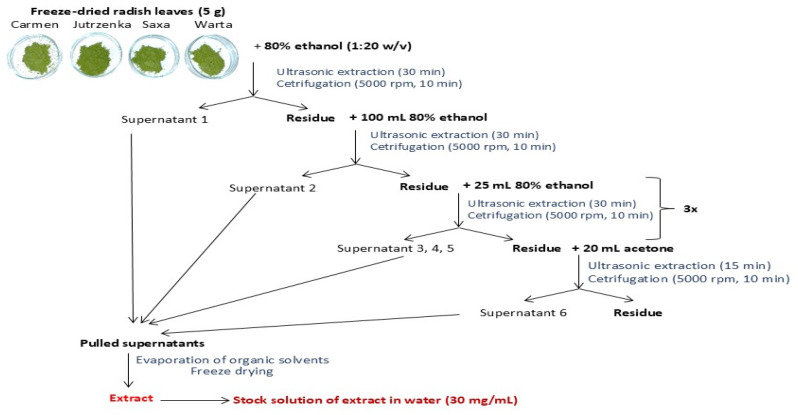
A graphical representation of leaf extract preparation.

Prior to the extraction, the dried leaves were crushed in a coffee grinder. The procedure for preparing leaf extracts is presented in Figure 5. Briefly, 80% ethanol and acetone were used as the extraction agents. The obtained aqueous solution of extractable compounds was freeze-dried after prior evaporation of the solvents (Rotavapor R-3, Büchi, Switzerland). For further analysis, working solutions of extracts were prepared at a concentration of 30 mg/mL.

### 4.4. Identification and Content of Individual Phenolic Compounds

For phenolic identification, crude extracts were additionally purified using a solid phase extraction method (SPE). In total, 4 mL of the extract was loaded onto a Sep-Pak C18 cartridge (2.5 g capacity, Waters Corp., Milford, MA, USA) that was previously activated with methanol (15 mL) and water (15 mL). The column was washed with water to eliminate carbohydrates, proteins, and other polar compounds. The phenolic compounds were eluted with methanol (15 mL), which was evaporated under reduced pressure (T < 40 °C) and dissolved in 2 mL of 50% methanol.

UPLC-MS analysis was performed on an ultra-performance liquid chromatograph (Waters Acquity UPLC system, Milford, MA, USA) equipped with a binary pump, an autosampler, a column compartment, and a diode array detector. Briefly, samples were eluted with a gradient of solvent A (4.5% formic acid in ultrapure water) and B (acetonitrile) on an Acquity UPLC HSS T3 C18 column (150 × 2.1 mm, 1.8 μm; Waters) operating at 30 °C, as described in the previous work [68]. The gradient program was as follows: initial conditions 99% (A), 3 min 75% (A), 10 min 60% (A), 12.5 min 100% (B), 15.0 min 99% (A). The flow rate was 0.45 mL/min, and the injection volume was 5 μL. The mass spectrometer was operating in the negative and positive mode for a mass range of 150–1500 Da, fixed source temperature at 100 °C, desolvation temperature 250 °C, desolvation gas flow of 600 L/h, cone voltage of 45 V, capillary voltage of 2.0 kV, and collision energy 50 V. Leucine enkephalin was used as a lock mass. The instrument was controlled by Mass-Lynx^TM^ V 4.1 software. The compounds were tentatively identified based on their UV–Vis spectra and MS and MS^2^ properties in comparison with the literature data.

A quantitative analysis of identified phenolics was based on the standards as follows: apigenin 7-glucoside was used for the apigenin derivatives, caffeic acid for caffeic acid derivatives, *p*-coumaric acid for *p*-coumaric acid and their glucoside, kaempferol 3-glucoside for kaempferol derivatives, sinapic acid for sinapic derivatives, ferulic acid for ferulic acid glucoside and feruoylmalic acid, and pelargonidin 3-glucoside for pelargonidin derivatives. The calibration curves are presented in Table S6. The results were expressed as mg per 100 g of dry weight.

### 4.5. Determination of Total Phenolics and Total Proanthocyanidins

The analysis of the total phenolic content was carried out using the modified Folin–Ciocalteu method. Briefly, 0.2 mL of diluted extract was mixed with 5 mL of water, 0.1 mL of Folin–Ciocalteu reagent, and with 1 mL of 20% sodium carbonate. The total volume of reaction mixture was made up to 10 mL with water. After 20 min of incubation, the absorbance of samples was read at 760 nm. The content of total phenolics was expressed as gallic acid equivalents per 100 g leaf dw (Table S6). The content of total proanthocyanidins was determined after their acid depolymerization to the corresponding anthocyanidins as described by Rösch et al [69]. In fact, 5–15 mg of freeze-dried leaves or 0.3–0.4 mL of leaf extracts were mixed with 10 mL of concentrated hydrochloric acid and n-butanol (1:9, *v/v*) mixture and heated for 90 min in a boiling-water bath. After cooling in ice, the absorbance of the sample was measured at 550 nm. The content of proanthocyanidins was expressed as mg of cyanidin equivalents/100 g dw of leaves.

### 4.6. Extraction and Analysis of Organic Acids and Vitamin C

Freeze-dried radish leaves (0.3 g) with 2 mL of 1% solution of meta-phosphoric acid were mixed for 2 min and centrifuged at 10,000 rpm for 10 min. The supernatant was filtered through syringe filters filled with regenerated cellulose (RC) with a pore size of 0.22 µm (Pureland, Chemland, Stargard, Poland). Individual organic acids in the supernatants were determined via the HPLC system with a photodiode array detector (Waters Corp., Milford, MA, USA). Separation was achieved on an ion exclusion Rezex ROA-Organic H+ column (300 *×* 7.8 mm, Phenomenex, Torrance, CA, USA) according to the procedure described by Aubert et al. [70]. The elution system was 0.005 N H_2_SO_4_, running isocratically at a flow rate 0.5 mL/min. Organic acids were quantified from the absorbance peaks at 210 nm and using calibration curves performed with citric, fumaric, malic, oxalic, succinic, and tartaric acid standards (Table S6). The results were expressed as mg of individual organic acid per 100 g dw of radish leaves.

Chromatographic determination of L-ascorbic acid was performed based on the work Medina-Lazano et. al. [71]. The UPLC analysis was carried out on an Acuality UPLC HSS T3 column (150 × 2.1 mm, 1.8 μm; Waters), and the mobile phases consisted of methanol (A) and ultrapure water pH 2.0 acidified with formic acid (B) with a flow rate of 0.3 mL/min of 2% A and 98% B in isocratic mode. The samples and column were kept at 5 and 30 °C, respectively. The injection volume was 5 µL, and the total running time was 3 min. The wavelength of the detector was set at 245 nm. The ascorbic acid content was quantified using a calibration curve (Table S6). The results were expressed as mg of L-ascorbic acid per 100 g dw.

### 4.7. Extraction and Determination of Chlorophyl and Carotenoid Pigments

Ground freeze-dried radish leaves (10 mg) with 2 mL of acetone (100%) were vortexed for 1 min and centrifuged at 10,000 rpm for 10 min. Another portion of acetone was added to the precipitate, and the process was repeated until the solvent was colorless. Absorbance of pooled acetone supernatants was measured at wavelengths 662 and 664 nm for chlorophyll a and b and 470 nm for carotenoids against acetone. The concentrations of chlorophyll a and b and total carotenoids were calculated using the equation described by Costache et al. [72] and expressed as mg/100 g dw.

### 4.8. Antioxidant Activity

The ability of radish leaves to scavenge ABTS radical cation, superoxide anion radical (SARSA method), and to reduce ferric ion (FRAP method) was investigated by the procedure described in our previous work [73]. The antioxidant activity determined by ABTS and FRAP methods was expressed as Trolox equivalents and, in the SARSA assay, as (+)-catechin equivalents per gram dw (Table S6). The ferrous-ion-chelating ability (FCA method) of the radish leaves was determined according to Cantele et al. [74]. Diluted extract (1 mL) was mixed with 20 µL of 2 mM FeCl_2_ and 20 µL of 6 mM ferrozine. After incubation for 10 min, the absorbance was read at 562 nm against the negative control (without ferrozine solution). The metal-chelating activity was expressed and EDTA equivalents per gram dw.

### 4.9. Proximate Analysis

The elementary chemical composition of leaves (dry matter, ash, fat, and protein contents) was determined using procedures described by Nollet [75]. Dry matter was estimated by drying at 105 °C to constant weight, the unit for the results was expressed in g/100 g of fresh leaves. Ash was determined through incineration of dried leaves in a muffle furnace at 600 °C for 6 h, crude fat by Soxhlet extraction with hexane, and crude protein via the Kjeldahl method (N × 6.25). The results were expressed in g/100 g of dw. The content of total carbohydrates was calculated on 100 g of leaves as the difference between 100 g dw and the sum of total fat, protein, and ash, expressed as g/100 g dw. Determination of total dietary fiber and its soluble and insoluble fraction was described in the previous study [73]. The total dietary fiber concentration was determined as the sum of soluble fiber (SDF) and insoluble fiber (IDF). The IDF concentration was the sum of neutral sugars, uronic acids, determined by spectrophotometric methods, and the mass of Klason lignins, which was quantified gravimetrically after subtracting the ash content. On the other hand, the concentration of SDF was the sum of uronic acids and neutral sugars. The content of total fiber, soluble fiber, and insoluble fiber was expressed as g/100 g dw.

### 4.10. In Vitro Digestion of Potato Starch

The simulated potato starch digestion was modified based on the methodology described by Kajszczak et al. [68]. The in vitro digestion process consisted of three stages: oral, gastric, and intestinal digestion, the course of which is described in Table 7. For the simulated digestion process, the following solutions were prepared: potato starch solution (0.5 g was gelatinized in 20 mL of water for 2.5 min from the moment of boiling. After cooling, the volume of the solution was made up to 20 mL with water) and α-glucosidase solution (20 mg acetone intestinal powder from rat with 1.2 mL of 0.9% NaCl solution extracted in an ultrasonic bath for 30 s in an ice bath, then 30 s without ultrasonic. This step was repeated 12 times. The mixture was centrifuged at 10,000 rpm for 10 min, and the supernatant was made up to a volume of 16 mL).

All digestion steps were carried out in a water-shaking bath. To determine the amount of glucose released from starch, after 120 min of simulated intestinal digestion, 0.5 mL of digestion and 0.5 mL 2M Tris-HCl buffer (pH 7.0) were taken into a tube containing 0.4 mL of a commercial glucose test (Biomaxima, Lublin, Poland). The samples were incubated at 37 °C for 10 min, after which the absorbance was measured at 500 nm. The IC_50_ values (concentration of the extract that caused 50% inhibition of starch hydrolysis) were calculated from a regression curve of the percentage (%) inhibition of glucose released against various concentration of leaf.

### 4.11. Glucose Binding Capacity

The determination of glucose-binding capacity was made based on the work of Jurevičiūtė, et al. [52]. To 50 mg of freeze-dried radish leaves, 1 mL of 10, 50, or 100 mM glucose solution were added. The samples were mixed on a vortex and incubated at 37 °C with constant shaking at 700 rpm for 6 h. Glucose solutions were also incubated as initial samples, as well as radish leaves with distilled water added instead of glucose to obtain reagent samples. After incubation, the samples were centrifuged for 10 min at 10,000 rpm and then diluted 50-fold. Glucose content was determined spectrophotometrically using the commercial glucose test (Biomaxima, Lublin, Poland), adding 1 mL of the diluted sample and 0.4 mL of the test. After incubation at 37 °C for 10 min, the absorbance was measured at 500 nm against the sample, which contained water and the Biomaxima test. The glucose-binding capacity was expressed as mmol of glucose binding per gram of radish leaves.

#### 4.12. α-Amylase Inhibition Assay

The α-amylase inhibition assay was based on a previously described spectrophotometric method, with some modifications [76]. All reagents were prepared in 0.1 M phosphate buffer containing 6 mM CaCl_2_ (pH 6.9). Briefly, the assay mixture consisted of 0.1 mL of stock extract solution (30 mg/mL), 0.2 mL of gelatinized potato starch (0.5 g/L), and 0.1 mL of α-amylase (13.3 µg/mL). After incubation at 37 °C for 10 min, the reaction was stopped by addition of 0.4 mL of 0.4 M HCl followed by 0.5 mL of 5 mM I_2_ in 5 mM KI. The absorbance was read at 600 nm. Absorbance of a control sample was measured without the leaf extract. Acarbose was used as a positive control. Each sample was measured in triplicate. The IC_50_ values (concentration of the extract that caused 50% inhibition) were calculated from a regression curve of the percentage inhibitions against various leaf extract concentrations.

#### 4.13. α-Glucosidase Inhibition Assay

The assessment of the α-glucosidase inhibitory activity was according to our previous work [76]. Briefly, 62.5 mg of rat intestinal acetone powder was mixed with 1.2 mL of 0.9% NaCl solution, and enzyme isolation was performed in an ultrasonic bath. The enzyme supernatant was poured and made up to a volume of 10 mL. The α- glucosidase reaction mixture contained 50 μL of enzyme supernatant (diluted twice for maltose and without dilution for sucrose as substrate) and 50 μL of diluted stock solution of leaf extract. After incubation at 37 °C for 10 min, 50 μL of maltose (0.1 M) or sucrose (0.5 M) was added and incubated at 37 °C for 20 min or 60 min for maltose and sucrose, respectively. The reaction was stopped by adding 150 µL of 2 M Tris–HCl buffer (pH 7.0). Acarbose was used as a positive control, while the control sample was prepared without extract. The concentrations of glucose released from the reaction mixtures were determined using the commercial glucose test (Biomaxima SA, Lublin, Poland). Each sample was measured in triplicate. The IC_50_ values were calculated from a regression curve of the percentage inhibitions against various leaf extract concentrations.

### 4.14. Protein Glycation Inhibition Assay

The bovine serum albumin (BSA) and glucose/fructose assay was carried according to the method of Kajszczak et al. with some modifications [76]. In total, 25 mL of the test solution containing D-glucose or D-fructose (1.0 M), BSA (10 mg/mL), and sodium azide (0.1 mg/mL) in the phosphate buffer (0.1 M, pH 7.4) were prepared. The test solution (0.5 mL) was incubated in the dark at 37 °C for 13 days for glucose and for 6 days for fructose with or without 0.25 mL of diluted stock solution of the extract. Next, the formation of AGEs was evaluated based on fluorescence intensity at λ = 330 nm (excitation wave) and λ = 410 nm (emission wave). The fluorescence intensity was measured in 96-well plates (black, OptiPlate-96 F, PerkinElmer, Ware, UK) in a microplate reader (Synergy2, BioTek Instruments Inc., Winooski, VT, USA). Aminoguanidine was used as a positive control. The IC_50_ values were calculated from a regression curve of the percentage inhibitions against various leaf extract concentrations.

### 4.15. Statistical Analysis

All samples were assayed in triplicate, and results are given as the mean ± standard deviation using Microsoft Excel XP. Significance differences were calculated via one-way analysis of variance (ANOVA) using Statistica Ver. 6.0 (TIBCO Software Inc., Palo Alto, CA, USA). Difference among means was determined by Tukey’s test at a significance level of *p* < 0.05. For comparison of the results of antidiabetic activity and composition of radish leaves, the coefficients of correlation were determined using a Pearson correlation test.

## 5. Conclusions

This is the first report in the literature on the anti-glucosidase and anti-glycation activity of radish leaves, as well as on the leaves’ effect on starch digestion and glucose-binding capacity. This study revealed that the radish leaves decrease glucose liberation during co-digestion with potato starch and bind free glucose due to the presence of dietary fiber and phenolic compounds like proanthocyanidins, flavones, and hydroxycinnamic acids. Moreover, radish leaf extracts exhibited potent inhibitory activity against α-glucosidase mostly due to the presence of proanthocyanidins and flavones. Therefore, the consumption of radish leaves, mostly discarded, may present nutritional value and health benefits due to their high content of protein, dietary fiber, chlorophyll pigments, and phenolic compounds, as well as antioxidant activity, demonstrated in various measurement systems. Therefore, radish leaves are attractive, unconventional sources of bioactive compounds and may be used to develop novel functional products with anti-diabetic activity, but more research is needed to confirm these properties. The question, which compound(s) present in radish leaves were responsible for the observed anti-diabetic properties, remains open.

## Figures and Tables

**Table 1:** Proximate analysis of radish leaves.

Compounds	Radish Cultivar			
	Carmen	Jutrzenka	Saxa	Warta
Dry matter (g/100 g fw)	7.94 ± 0.18 ^a^	8.56 ± 0.12 ^bc^	8.80 ± 0.16 ^c^	8.36 ± 0.15 ^b^
Ash (g/100 g dw)	24.19 ± 0.12 ^c^	21.03 ± 0.41 ^a^	22.97 ±0.16 ^bc^	21.98 ± 0.88 ^ab^
Protein (g/100 g dw)	21.47 ± 0.09 ^a^	22.31 ± 0.10 ^b^	22.49 ± 0.50 ^b^	22.60 ± 0.15 ^b^
Fat (g/100 g dw)	5.32 ± 0.07 ^a^	8.19 ± 0.07 ^d^	6.14 ± 0.06 ^c^	5.73 ± 0.10 ^b^
Carbohydrates * (g/100 g dw)	49.02 ± 0.18 ^ab^	48.18 ± 0.54 ^a^	48.40 ± 0.63 ^a^	49.98 ± 0.83 ^b^
Dietary fiber (g/100 g dw)	41.07 ± 1.21 ^a^	41.64 ± 1.21 ^ab^	42.98 ± 0.38 ^ab^	44.39 ± 1.80 ^b^
Insoluble dietary fiber (g/100 g dw)	39.31 ± 1.16 ^a^	40.06 ± 1.19 ^ab^	41.56 ± 0.39 ^ab^	43.08 ± 1.80 ^b^
Soluble dietary fiber (g/100 g dw)	1.76 ± 0.06 ^b^	1.59 ± 0.05 ^b^	1.42 ± 0.03 ^a^	1.31 ± 0.08 ^a^
Total organic acids (g/100 g dw)	0.85 ± 0.02 ^c^	0.71 ± 0.01 ^a^	0.77 ± 0.02 ^b^	0.86 ± 0.03 ^c^

* Calculated as 100 − (protein + fat + ash). Data corresponds to the average ± standard deviation of three replicates. fw—fresh weight, dw—dry weight. Different letters superscripts in the same row indicate significant difference (*p* < 0.05).

**Table 2:** Content (mg/100 g dw) of bioactive compounds of radish leaves.

Compounds	Radish Cultivar			
	Carmen	Jutrzenka	Saxa	Warta
Total phenolics	955.83 ± 39.68 ^a^	1386.59 ± 56.41 ^c^	1124.90 ± 45.58 ^b^	1367.65 ± 61.75 ^c^
Total proanthocyanidins	226.92 ± 7.51 ^ab^	207.87 ± 12.24 ^a^	233.82 ± 8.76 ^b^	263.94 ± 2.83 ^c^
Total chlorophyll	567.28 ± 1.56 ^a^	665.40 ± 13.23 ^b^	642.18 ± 0.41 ^b^	555.76 ± 12.99 ^a^
Chlorophyll *a*	387.03 ± 4.95 ^a^	470.39 ± 18.77 ^b^	439.90 ± 2.73 ^b^	382.82 ± 15.24 ^a^
Chlorophyll *b*	180.26 ± 2.05 ^ab^	195.01 ± 13.47 ^b^	202.28 ± 7.82 ^b^	171.95± 7.41 ^a^
Total carotenoids	55.64 ± 2.94 ^a^	71.35 ± 5.53 ^b^	60.58 ± 2.40 ^ab^	59.16 ± 4.98 ^a^
L-ascorbic acid	635.22 ± 61.18 ^a^	683.14 ± 36.59 ^a^	563.32 ± 46.30 ^a^	630.81 ± 73.02 ^a^

Data correspond to the average ± standard deviation of six replicates for total phenolics and from three replicates for the remaining compounds. Different letters superscripts in the same row indicate a significant difference (*p* < 0.05).

**Table 3:** Antioxidant capacity of radish leaves determined by different methods.

Method	Radish Cultivar			
	Carmen	Jutrzenka	Saxa	Warta
ABTS	3.10 ± 0.12 ^a^	5.31 ± 0.40 ^c^	4.16 ± 0.27 ^b^	5.18 ± 0.32 ^c^
SARSA	24.33 ± 2.01 ^b^	42.55 ± 3.66 ^c^	19.13 ± 1.81 ^a^	22.96 ± 1.87 ^ab^
FRAP	3.47 ± 0.11 ^a^	5.98 ± 0.28 ^c^	4.48 ± 0.16 ^b^	5.75 ± 0.33 ^c^
FCA	0.51 ± 0.08 ^a^	1.86 ± 0.10 ^c^	0.75 ± 0.09 ^b^	0.67 ± 0.08 ^b^

Data correspond to the average ± standard deviation of six replicates. The same letters superscripts indicate no significant differences within the row (*p* < 0.05). ABTS and FRAP are expressed as mmol Trolox, SARSA as mmol (+)-catechin, and FCA as mg EDTA equivalents per gram dw.

**Table 4:** Content (mg/100 g dw) the individual phenolic compounds of radish leaves.

Phenolic Compound	Radish Cultivar			
	Carmen	Jutrzenka	Saxa	Warta
Caffeic acid glucoside	6.49 ± 0.54 ^a^	44.46 ± 4.01 ^c^	20.37 ± 2.51 ^b^	65.94 ± 8.01 ^d^
*p*-Coumaric acid glucoside	6.12 ± 0.25 ^b^	19.92 ± 1.70 ^c^	2.35 ± 0.10 ^a^	5.81 ± 0.42 ^b^
Sinapic acid glucoside	1.56 ± 0.14 ^a^	4.84 ± 0.78 ^c^	3.09 ± 0.11 ^b^	3.94 ± 0.40 ^bc^
Ferulic acid glucoside	0.65 ± 0.02 ^a^	3.52 ± 0.15 ^d^	1.10 ± 0.06 ^b^	1.89 ± 0.05 ^c^
1,2-Disinapoylglucoside	-	24.56 ± 2.79 ^b^	10.25 ± 0.35 ^a^	36.80 ± 3.34 ^c^
Feruloylmalic acid	-	40.52 ± 0.86 ^c^	30.95 ± 1.61 ^b^	16.79 ± 2.82 ^a^
*p*-Coumaric acid	-	-	-	72.84 ± 6.29
Sum of hydroxycinnamic acids	14.82 ± 0.95 ^a^	137.82 ± 9.18 ^c^	68.11 ± 4.48 ^b^	204.01 ± 18.06 ^d^
Kaempferol 3-diglucoside	-	2.29 ± 0.18 ^a^	-	10.60 ± 0.84 ^b^
Kaempferol 3-*O*-coumaroyl glucoside	2.75 ± 0.02 ^a^	4.11 ± 0.40 ^b^	-	2.61 ± 0.26 ^a^
Kaempferol-3-*O*-glucosyl-rhamnosyl-glucoside	24.43 ± 0.84 ^a^	106.78 ± 4.68 ^c^	28.18 ± 1.36 ^a^	42.05 ± 2.74 ^b^
Kaempferol 3-*O*-coumaroyl glucoside	282.62 ± 6.19 ^a^	430.84 ± 7.80 ^c^	357.50 ± 9.42 ^b^	429.84 ± 18.43 ^c^
kaempferol-3-*O*-arabinoside-7-*O*-rhamnoside	162.71 ± 0.69 ^b^	161.44 ± 13.75 ^b^	130.95 ± 3.49 ^a^	198.15 ± 18.29 ^c^
Kaempferol 3-*O*-rhamnoside-7-*O*-rutinoside	17.54 ± 0.36 ^b^	-	15.05 ± 0.71 ^a^	18.68 ± 1.11 ^b^
Kaempferol 3-(*p*-coumaroyl)sophorotrioside	179.65 ± 4.85 ^a^	502.53 ± 19.29 ^d^	317.78 ± 23.15 ^c^	215.40 ± 19.25 ^b^
Kaempferol 3-*O*-(*p*-coumaroyl)dirhamnosylhexoside	190.57 ± 4.43 ^a^	325.95 ± 16.47 ^d^	223.23 ± 7.67 ^a^	283.57 ± 23.15 ^b^
Kaempferol 3-*O*-rutionoside-7-*O*-rhamnoside	-	48.93 ± 5.18	-	-
Kaempferol 3-*O*-*p*-coumaryl rutinoside-7-*O*-arabinoside	-	25.70 ± 1.96	-	-
Sum of flavonols	860.27 ± 17.39 ^a^	1608.57 ± 69.71 ^d^	1072.69 ± 45.00 ^b^	1200.90 ± 13.20 ^c^
Apigenin-C-hexoside-C-pentoside	-	5.28 ± 0.55 ^c^	1.74 ± 0.16 ^a^	3.22 ± 0.52 ^b^
Apigenin-7-*O*-rutinoside	202.79 ± 4.84 ^a^	180.16 ± 23.33 ^a^	186.25 ± 2.96 ^a^	296.82 ± 15.82 ^b^
Sum of flavones	202.79 ± 4.84 ^a^	185.44 ± 23.87 ^a^	187.99 ± 3.12 ^a^	300.04 ± 16.35 ^b^
Pelargonidin -3-(feruloyl)diglucoside-5-(malonyl)glucoside	5.27 ± 0.23 ^b^	-	6.61 ± 0.28 ^c^	4.10 ± 0.12 ^a^
Pelargonidin -3-(feruloyl)diglucoside-5-glucoside derivative	3.52 ± 0.25 ^a^	-	3.22 ± 0.10 ^a^	3.40 ± 0.17 ^a^
Pelargonidin -3-(*p*-coumaroyl)diglucoside-5-glucoside derivative	2.80 ± 0.27	-	-	-
Sum of antocyanins	11.59 ± 0.75 ^c^	-	9.83 ± 0.38 ^b^	7.50 ± 0.05 ^a^
Total phenolics	1089.47 ± 32.13 ^a^	1931.83 ± 50.29 ^d^	1338.62 ± 51.74 ^b^	1712.45 ± 18.11 ^c^

Data correspond to the average ± standard deviation of three replicates, and different letters superscripts within the same row indicate statistically significant differences at *p* < 0.05 between cultivars. The content of flavones was expressed as apigenin 7-glucoside equivalents, anthocyanins as pelargonidin 3-glucoside, kaempferol derivatives as kaempferol 3-glucoside, caffeic acid derivatives as caffeic acid, *p*-coumaric acid derivatives as *p*-coumaric acid, sinapic acid derivatives as sinapic acid, and ferulic acid derivatives as ferulic acid.

**Table 5:** Glucose-binding capacity of radish leaves.

Radish Cultivar	Glucose Concentration in the Solution		
	10 mM	50 mM	100 mM
	Amount of Glucose Binding [mmol/g of Leaf)		
Carmen	0.10 ± 0.00 ^b^	0.18 ± 0.02 ^bc^	0.31 ± 0.01 ^ab^
Jutrzenka	0.08 ± 0.00 ^a^	0.11 ± 0.02 ^a^	0.29 ± 0.01 ^a^
Saxa	0.08 ± 0.00 ^a^	0.13 ± 0.01 ^ab^	0.28 ± 0.01 ^a^
Warta	0.10 ± 0.01 ^b^	0.21 ± 0.02 ^c^	0.34 ± 0.01 ^b^

The results are reported as mean ± standard deviations (*n* = 3). Different letters superscripts indicate significant differences within the column (*p* < 0.05).

**Table 6:** Content (mg/g extract) of different phenolic compounds of radish leaf extracts.

Sub-Group of Phenolic Compounds	Carmen	Jutrzenka	Saxa	Warta
	mg/g of Extract			
Total proanthocyanidins	0.145 ± 0.003 ^b^	0.112 ± 0.002 ^a^	0.216 ± 0.003 ^c^	0.208 ± 0.002 ^c^
Total hydroxycinnamic acids	1.28 ± 0.06 ^a^	10.57 ± 0.73 ^c^	6.67 ± 0.42 ^b^	17.72 ± 0.96 ^d^
Total flavonols	74.20 ± 1.37 ^a^	104.96 ± 1.23 ^c^	84.94 ± 3.61 ^b^	86.98 ± 0.05 ^b^
Total flavones	17.49 ± 0.42 ^b^	12.10 ± 1.55 ^a^	15.95 ± 0.24 ^b^	21.76 ± 1.18 ^c^
Total anthocyanins	0.65 ± 0.02 ^a^	0.00	0.86 ± 0.03 ^b^	0.83 ± 0.04^b^
Total phenolics	93.61 ± 1.78 ^a^	126.03 ± 3.04 ^c^	107.56 ± 4.24 ^b^	124.63 ± 1.89 ^c^

Data correspond to the average ± standard deviation of three replicates, and different letters superscripts within the same row indicate statistically significant differences at *p* < 0.05 between cultivars.

**Table 7:** Composition of the mixtures of the three-stages-simulated in vitro digestion of potato starch.

Oral digestion; Incubation conditions: 37 °C, 2 min
10–50 mg of freeze-dried radish leaves 0.5 mL of water 0.5 mL of gelatinized potato starch (25 mg/mL) 1.25 mL saliva solution 0.25 mL α-amylase solution (0.1 mg/mL)
Gastric digestion; Incubation conditions: 37 °C, 2 h
2.25 mL gastric solution (2 g NaCl in 0.7% HCl in water, pH 1.2) 0.25 mL pepsin solution (3.2 mg/mL) pH correction to a value of 2.0 with 2M NaOH
Intestinal digestion; Incubation conditions: 37 °C, 2 h
2.5 mL of water pH correction to a value of 6.0 with 2M NaOH followed to 7.5 with 1M NHCO_3_ The volume of the sample was adjusted to 8.2 mL with water 0.5 mL of bile salts (100 mg/mL) 1 mL of α-glucosidase solution 0.3 mL of pancreatin solution (0.04 mg/mL)

## Data Availability

Data are contained within the article and Supplementary Materials.

## Supplementary Materials

The following supporting information can be downloaded at https://www.mdpi.com/article/10.3390/molecules29235689/s1, Figure S1: UPLC chromatograms of phenolic compounds of four radish leaf cultivars; Table S1: The content of organic acids in radish leaves (mg/100 g dry weight); Table S2: UPLC-ESI-Q-TOF/MS data and tentative identification of phenolic compounds of radish leaves; Table S3: Pearson’s correlation coefficients between inhibition of potato starch digestion, glucose-binding capacity and content of macronutrients and phenolic compounds; Table S4: Content (mg/g extract) of the individual phenolic compounds of radish leaf extracts; Table S5: Pearson’s correlation coefficients (*r*) between inhibition of α-glucosidase and AGEs formation and phenolic compound content of radish leaf extracts. Table S6: Calibration curves determined in the research methods.
