# Supplementary material for: Composition, Anti-Diabetic, and Antioxidant Potential of Raphanus sativus Leaves
Source: Molecules. 2024 Dec 1;29(23):5689. doi: 10.3390/molecules29235689 (PMC11643074; doi:10.3390/molecules29235689)
Supplement: Supplementary file 1 [file molecules-29-05689-s001.zip › molecules-3313577-supplementary.pdf]

Composition, anti-diabetic and antioxidant potential of *Raphanus sativus* leaves

Dominika Kajszczak, Dorota Sosnowska, Barbara Frąszczak and Anna Podsędek

Table S1. The content of organic acids in radish leaves (mg/100 g dry weight)

Compounds	Carmen	Jutrzenka	Saxa	Warta
Citric acid	283.43 ± 11.00[c]	197.10 ± 5.16[a]	216.55 ± 13.59[a,b]	227.16 ± 3.24[b]
Fumaric acid	2.56 ± 0.09[a]	3.37 ± 0.04[b]	4.05 ± 0.28[c]	3.64 ± 0.24[b,c]
Malic acid	113.38 ± 7.10[c]	71.55 ± 2.53[a]	98.01 ± 4.90[b]	83.24 ± 1.44[a]
Oxalic acid	189.36 ± 5.80[a]	215.96 ± 4.16[b]	205.32 ± 10.81[a,b]	226.38 ± 13.74[b]
Succinic acid	247.72 ± 1.81[a]	246.46 ± 7.66[a]	244.12 ± 1.67[a]	322.91 ± 15.55[b]
Tartaric acid	16.23 ± 0.36[b,c]	15.46 ± 0.40[a,b]	17.02 ± 0.73[c]	14.04 ± 0.75[a]
Total	849.35 ± 21.65[b]	749.90 ± 13.36[a]	784.98 ± 19.02[a]	877.37 ± 27.79[b]

Data corresponds to the average ± standard deviation of three replicates. Different letters superscripts in the same row indicate significant difference ($p < 0.05$).

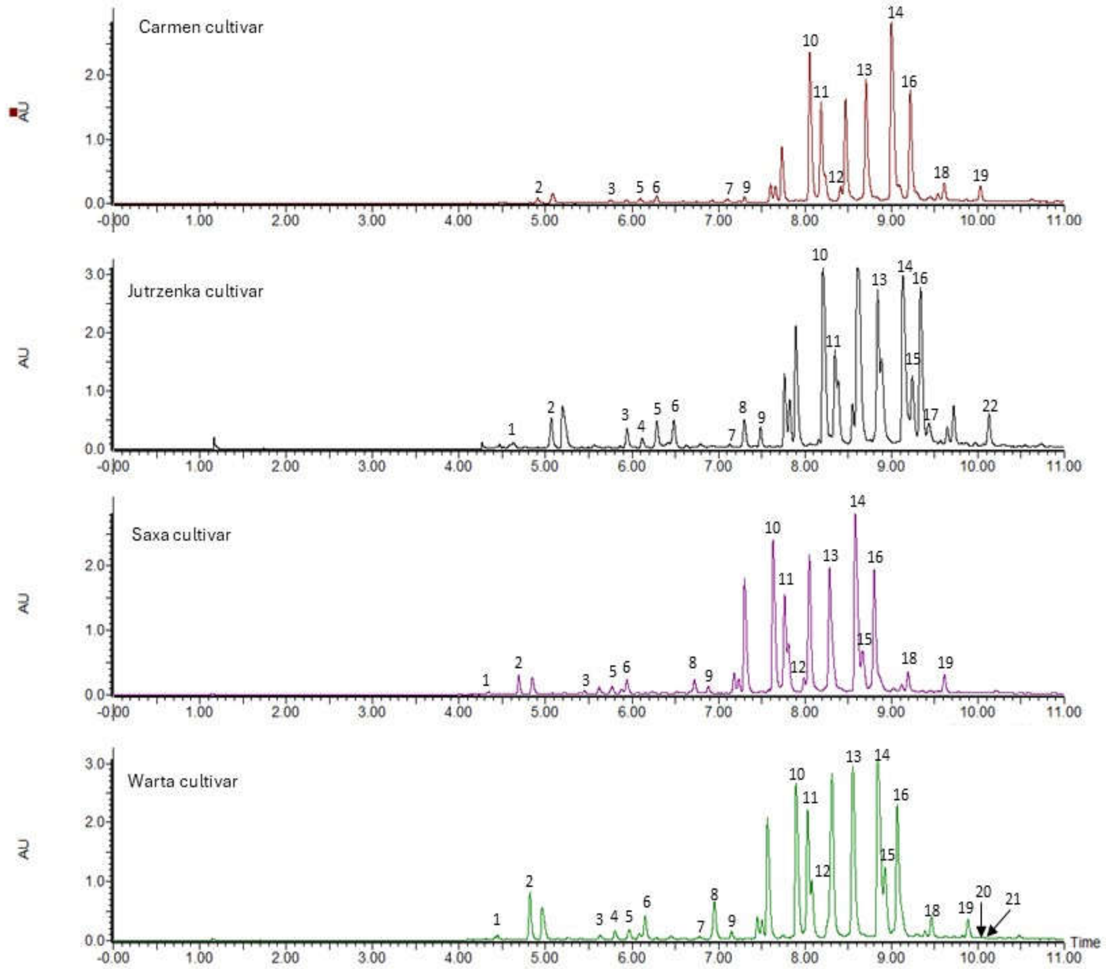

Figure S1. UPLC chromatograms of phenolic compounds of four radish leaf cultivars. Refer to Table S2 for the identification of peaks.

Table S2. UPLC-ESI-Q-TOF/MS data and tentative identification of phenolic compounds of radish leaves.

No.	R_t (min)	λ_{max} (nm)	$[M-H]^-$ /$[M+H]^+$ (m/z)	MS/MS (m/z)	Tentative identification	Ref.
1	4.21-4.80	319	563/-	353,193,175,473	Apigenin-C-hexoside-C-pentoside	[1]
2	4.60-5.23	329	341/-	161/135/179	Caffeic acid glucoside	[2]
3	5.40-6.00	315	325/-	145/325/163/119	*p*-Coumaric acid glucoside	[2]
4	5.71-6.20	369	609/-	447/285	Kaempferol 3-diglucoside	[3]
5	5.70-6.43	328	385/-	205/190/175/223	Sinapic acid glucoside	[2]
6	5.91-6.61	324	355/-	175/161/193/135	Ferulic acid glucoside	[2]
7	6.59-7.20	338	593/-	285/447/594/430	Kaempferol 3-*O*-*p*-coumaroyl glucoside	[4]
8	6.60-7.41	328	591/-	133/223/179/367	1,2-Disinapoylglucoside	[3]
9	6.84-7.67	334	755/-	431/285/447/593	Kaempferol-3-*O*-glucosyl-rhamnosyl-glucoside	[5]
10	7.51-8.31	338	593/-	285/447/594/430	Kaempferol 3-*O*-coumaroyl glucoside	[4]
11	7.72-8.55	334	563/-	417/431/564/285	Kaempferol-3-*O*-arabinoside-7-*O*-rhamnoside	[4]
12	8.04-8.41	341	785/-	431/739/285/740	Kaempferol 3-*O*-rhamnoside-7-*O*-rutinoside	[4]
13	8.27-9.05	332	917/-	469/755/477/285	Kaempferol 3-(*p*-coumaroyl)sophorotrioside	[3]
14	8.53-9.23	310	577/-	285/577/431/578	Apigenin-7-*O*-rutinoside	[5]
15	8.63-9.34	327	309/-	193/133/194/178	Feruloylmalic acid	[3]
16	8.72-9.52	337	901/-	447/287/755/431	Kaempferol 3-*O*-(*p*-coumaroyl)dirhamnosylhexoside	[6]
17	9.26-9.46	340	739/-	163/431/285/593	Kaempferol 3-*O*-rutionoside-7-*O*-rhamnoside	[4]
18	9.38-9.31	509	-/1019	271/519/771	Pelargonidin -3-(feruloyl)diglucoside-5-(malonyl)glucoside	[7]
19	9.55-9.99	514	-/1313	271/771/933	Pelargonidin -3-(feruloyl)diglucoside-5-glucoside derivative	[7]
20	9.86-10.12	309	163/-	119/163	*p*-Coumaric acid	[8]
21	9.97-10.30	515	-/1343	271/741/903	Pelargonidin -3-(p-coumaroyl)diglucoside-5-glucoside derivative	[7]
22	10.01-10.14	339	871/-	285/417/739/453	Kaempferol 3-*O*-*p*-coumaryl rutinoside-7-*O*-arabinoside	[4]

Table S3. Pearson's correlation coefficients between inhibition of potato starch digestion, glucose binding capacity and content of macronutrients and phenolic compounds.

	Inhibition of starch digestion (IC_{50} mg/mL)	Glucose binding capacity (mmol/g)
Dietary fiber	-0.781	0.509
Insoluble dietary fiber	-0.790	0.486
Soluble dietary fiber	0.821	-0.300
Protein	-0.866	0.036
Fat	-0.344	-0.486
Total phenolics[1]	-0.934	0.227
Proanthocyanidins[1]	-0.469	0.762
Total phenolics[2]	-0.831	0.149
HCA[2]	-0.987	0.527
Flavonols[2]	-0.640	-0.185
Flavones[2]	-0.647	0.932
Anthocyanins[2]	-0.083	0.487

1- determined by spectrophotometric method, 2- determined by UPLC analysis.

Table S4. Content (mg/g extract) of the individual phenolic compounds of radish leaf extracts.

Phenolic compounds	Carmen	Jutrzenka	Saxa	Warta
Caffeic acid glucoside	0.56 ± 0.05[a]	2.90 ± 0.26[c]	1.73 ± 0.21[b]	4.78 ± 0.58[d]
p-Coumaric acid glucoside	0.53 ± 0.02[b]	1.30 ± 0.11[c]	0.22 ± 0.04[a]	0.42 ± 0.03[b]
Sinapic acid glucoside	0.13 ± 0.01[a]	0.32 ± 0.05[b]	0.26 ± 0.01[b]	0.25 ± 0.04[b]
Ferulic acid glucoside	0.06 ± 0.00[a]	0.20 ± 0.06[c]	0.09 ± 0.01[a,b]	0.14 ± 0.00[b,c]
1,2-Disinapoylglucoside	-	1.60 ± 0.18[b]	0.87 ± 0.03[a]	2.67 ± 0.24[c]
Feruloylmalic acid	-	2.64 ± 0.06[b]	2.60 ± 0.14[b]	1.52 ± 0.72[a]
p-Coumaric acid	-	-	-	5.28 ± 0.56
Sum of hydroxycinnamic acids	1.28 ± 0.06[a]	10.57 ± 0.73[c]	6.67 ± 0.42[b]	17.72 ± 0.96[d]
Kaempferol 3-diglucoside	-	0.15 ± 0.01[a]	-	0.72 ± 0.05[b]
Kaempferol 3-*O*-coumaroyl glucoside	0.24 ± 0.00[a]	0.20 ± 0.03[a]	-	0.19 ± 0.05[a]
Kaempferol 3-*O*-glucosyl-rhamnosyl-glucoside	2.11 ± 0.07[a]	6.97 ± 0.31[c]	2.39 ± 0.12[a]	3.05 ± 0.20[b]
Kaempferol 3-*O*-coumaroyl glucoside	24.38 ± 0.53[a]	28.11 ± 0.51[b]	30.34 ± 0.80[b,c]	31.17 ± 1.34[c]
Kaempferol-3-*O*-arabinoside-7-*O*-rhamnoside	14.03 ± 0.06[b]	10.53 ± 0.90[a]	11.11 ± 0.30[a]	14.37 ± 1.33[b]
Kaempferol 3-*O*-rhamnoside-7-*O*-rutinoside	1.51 ± 0.03[b]	-	1.28 ± 0.06[a]	1.35 ± 0.08[a]
Kaempferol 3-(*p*-coumaroyl)-sophorotrioside	15.49 ± 0.42[a]	32.79 ± 1.26[c]	26.97 ± 1.96[b]	15.52 ± 0.24[a]
Kaempferol 3-*O*-(*p*-coumaroyl)dirhamnosylhexoside	16.44 ± 0.38[b]	21.27 ± 1.07[c]	12.85 ± 0.44[a]	20.56 ± 1.68[c]
Kaempferol 3-*O*-rutionoside-7-*O*-rhamnoside	-	3.19 ± 0.34	-	-
Kaempferol 3-*O*-coumaryl rutinoside-7-*O*-arabinoside	-	1.68 ± 0.13	-	-
Sum of flavonols	74.20 ± 1.37[a]	104.96 ± 1.23[c]	84.94 ± 3.61[b]	86.98 ± 0.05[b]
Apigenin 6-C-arabinoside-8-C-glucoside	-	0.34 ± 0.04[c]	0.15 ± 0.01[a]	0.23 ± 0.04[b]
Apigenin-7-*O*-rutinoside	17.49 ± 0.42[b]	11.76 ± 1.52[a]	15.81 ± 0.25[b]	21.53 ± 1.15[c]
Sum of flavones	17.49 ± 0.42[b]	12.10 ± 1.55[a]	15.95 ± 0.24[b]	21.76 ± 1.18[c]
Pelargonidin 3-(feruloyl)diglucoside-5-(malonyl)glucoside	0.35 ± 0.01[a]	-	0.56 ± 0.02[b]	0.38 ± 0.02[a]
Pelargonidin 3-(feruloyl)diglucoside-5-glucoside derivative	0.29 ± 0.01[a]	-	0.30 ± 0.05[a]	0.24 ± 0.04[a]
Pelargonidin 3-(p-coumaroyl)diglucoside-5-glucoside derivative	-	-	-	0.20 ± 0.02
Sum of anthocyanins	0.65 ± 0.02[b]	0.00[a]	0.86 ± 0.03[c]	0.83 ± 0.04[c]
Total phenolics	93.61 ± 1.78[a]	126.03 ± 3.04[c]	107.56 ± 4.24[b]	124.63 ± 1.89[c]

Data corresponds to the average ± standard deviation of three replicates and different letters superscripts within the same row indicate statistically significant differences at $p < 0.05$ between cultivars. The content of flavones was expressed as apigenin 7-glucoside equivalents, anthocyanins as pelargonidin 3-glucoside, kaempferol derivatives as kaempferol 3-glucoside, caffeic acid derivatives as caffeic acid, *p*-coumaric acid derivatives as *p*-coumaric acid, sinapic acid derivatives as sinapic acid, and ferulic acid derivatives as ferulic acid.

Table S5. Pearson's correlation coefficients (*r*) between inhibition of α-glucosidase and AGEs formation and phenolic compound content of radish leaf extracts.

	Inhibition of α-glucosidase - maltose (IC_{50} mg/mL)	Inhibition of α-glucosidase - sucrose (IC_{50} mg/mL)	AGEs formation in the presence of glucose (IC_{50} mg/mL)	AGEs formation in the presence of fructose (IC_{50} mg/mL)
Proanthocyanidins[1]	-0.921	0.210	0.443	-0.720
Total phenolics[2]	-0.175	0.517	-0.840	-0.356
HCA[2]	-0.573	0.785	-0.501	-0.395
Flavonols[2]	0.3.46	0.033	-1.000	-0.224
Flavones[2]	-0.854	0.728	0.592	-0.039
Anthocyanins[2]	-0.824	0.175	0.787	-0.375

1- determined by spectrophotometric method, 2- determined by UPLC analysis.

Table S6. Calibration curves determined in the research methods.

Compound	Calibration curve	Concentration range	Correlation coefficient
Apigenin 7-glucoside	Peak area = 476285 *(mg/mL)	0.04–0.33 mg/mL	0.9991
Kaempferol 3-glucoside	Peak area = 323033 *(mg/mL)	0.04–0.67 mg/mL	0.9907
Pelargonidin 3-glucoside	Peak area = 105360 *(mg/mL)	0.25–1.00 mg/mL	0.9657
Caffeic acid	Peak area = 543462 *(mg/mL)	0.06–0.50 mg/mL	0.9839
p-Coumaric acid	Peak area = 793784 *(mg/mL)	0.08–0.75 mg/mL	0.9570
Sinapic acid	Peak area = 937417 *(mg/mL)	0.04–0.33 mg/mL	0.9997
Ferulic acid	Peak area = 1016500 *(mg/mL)	0.04–0.33 mg/mL	0,9988
Gallic acid	Absorbance = 0.098 *(μg/mL)	1–8 μg/mL	0.9988
L-ascorbic acid	Peak area = 738.73 *(μg/mL)	0.5–100 μg/mL	0.9891
Trolox (ABTS method)	% Reduction = 4.1372 *(μM Trolox)	0.5–17 μM	0.9892
Trolox (FRAP method)	Absorbance = 0.0534 *(μM Trolox)	0.5–15 μM	0.9986
(+)-Catechin (SARSA method)	Absorbance = 139.15 *(mM (+)-catechin)	0.08–0.55	0.9740
EDTA (FCA method)	Chelating activity (%) = 35.596*(μg EDTA)	0.3–2.0 μg/mL	0.9931
Citric acid	Peak area = 1271.2 *(mg/mL)	0.30–10 mg/mL	0.9994
Fumaric acid	Peak area = 86431 *(mg/mL)	0.08–10 mg/mL	0.9922
Malic acid	Peak area = 933.98 *(mg/mL)	0.30–10 mg/mL	0.9999
Oxalic acid	Peak area = 13974 *(mg/mL)	0.08–10 mg/mL	0.9979
Succinic acid	Peak area = 658.03 *(mg/mL)	0.30–10	0.9986
Tartaric acid	Peak area = 1774.4 *(mg/mL)	0.30–10	0.9998

References

1. Cattivelli, A.; Zannini, M.; Conte, A.; Tagliazucchi, D. Inhibition of starch hydrolysis during *in vitro* co-digestion of pasta with phenolic compound-rich vegetable foods. *Food Biosci.* **2024**, *61*, 104586.
2. Sabry, M.M.; El-Halawany, A.M.; Fahmy, W.G.; Eltanany, B.M.; Pont, L.; Benavente, F.; Attia, A.S.; Sherbiny, F.F.; Ibrahim, R.M. Evidence on the inhibitory effect of Brassica plants against *Acinetobacter baumannii* lipases: Phytochemical analysis, *in vitro*, and molecular docking studies. *BMC Complement. Med. Ther.* **2024**, *24*, 164.
3. Lin, L.-Z.; Sun, J.; Chen, P.; Harnly, J.A. LC-PDA-ESI/MS^n identification of new anthocyanins in purple bordeaux radish (*Raphanus sativus* L. Variety). *J. Agric. Food Chem.* **2011**, *59*, 6616–6627.
4. Farid, M.M.; Ibrahim, F.M.; Ragheb, A.Y.; Mohammed, R.S.; Hegazi, N.M.; Shabrawy, M.O.EL; Kawashty, S.A.; Marzouk, M.M. Comprehensive phytochemical characterization of *Raphanus raphanistrum* L.: *In vitro* antioxidant and antihyperglycemic evaluation. *Sci. Afr.* **2022**, *16*, e01154.

5. Koley, T.K.; Khan, Z.; Oulkar, D.; Singh, B.K.; Maurya, A.; Singh, B.; Banerjee, K. High resolution LC-MS characterization of phenolic compounds and the evaluation of antioxidant properties of a tropical purple radish genotype. *Arab. J. Chem.* **2020**, *13*, 1355-1366.
6. Zhao, Y.; Chen, P.; Lin, L.; Harnly, J.M.; Yu, L.L.; Li, Z. Tentative identification, quantitation, and principal component analysis of green pu-erh, green, and white teas using UPLC/DAD/MS. *Food Chem.* **2011**, *126*, 1269-1277.
7. Jing, P.; Zhao, S.-J.; Ruan, S.-Y.; Xie, Z.-H.; Dong, Y.; (Lucy) Yu, L. Anthocyanin and glucosinolate occurrences in the roots of Chinese red radish (*Raphanus sativus* L.), and their stability to heat and pH. *Food Chem*. **2012**, *133*, 1569–1576.
8. Ibrahim, R.M.; Fayez, S.; Eltanany, B.M.; Abu-Elghait, M.; El-Demerdash, A.; Badawy, M.S.E.; Pont, L.; Benavente F.; Saber, F.R. Agro-byproduct valorization of radish and turnip leaves and roots as new sources of antibacterial and antivirulence agents through metabolomics and molecular networking. *Sci. Hortic.* **2024**, *328*, 112924.
